# Clinical features, prognostic factors, and survival of patients with antisynthetase syndrome and interstitial lung disease

**DOI:** 10.3389/fimmu.2022.872615

**Published:** 2022-08-10

**Authors:** Na Zhao, Wei Jiang, Hongliang Wu, Ping Wang, Xiaoni Wang, Yu Bai, Yao Li, Yanchun Tang, Ying Liu

**Affiliations:** ^1^ Department of Rheumatology, The Affiliated Yantai Yuhuangding Hospital of Qingdao University, Yantai, China; ^2^ Department of Radiotherapy, The Affiliated Yantai Yuhuangding Hospital of Qingdao University, Yantai, China; ^3^ Department of Neurology, The Affiliated Yantai Yuhuangding Hospital of Qingdao University, Yantai, China; ^4^ Department of Radiology, The Affiliated Yantai Yuhuangding Hospital of Qingdao University, Yantai, China

**Keywords:** interstitial lung disease, antisynthetase syndrome, prognostic factors, clinical features, survival factors

## Abstract

**Objective:**

This study aimed to analyze the clinical features and prognostic factors of imaging progression and survival in patients with antisynthetase syndrome (ASS) complicated by interstitial lung disease (ILD) in a large Chinese cohort.

**Methods:**

Medical records, imaging, and serological data of 111 patients with ASS-ILD (positive for at least one of the following autoantibodies: anti-Jo1, anti-PL7, anti-PL12, and anti-EJ) from the Affiliated Yantai Yuhuangding Hospital of Qingdao University database were retrospectively investigated. According to the changes in high-resolution computed tomography (HRCT) outcomes at 1 year follow-up, Patients were categorized into three groups: the regression, stability, and deterioration groups. Univariate analysis was performed to evaluate the possible prognostic factors of ILD outcome and death, and multivariate analysis was performed to determine the independent predictors of ASS-ILD outcome and death by logistic regression.

**Results:**

The number of CD3-CD19+ cells and initial glucocorticoid dosage were correlated with imaging progression, and may be independent risk factors for ILD deterioration. Dyspnea as the first symptom, hypohemoglobinemia, the serum ferritin level, oxygen partial pressure at diagnosis, and different treatment types were important factors affecting survival, and the initial serum ferritin level may be an independent risk factor for survival.

**Conclusions:**

The clinical characteristics of patients with ASS-ILD with different antisynthetase antibody subtypes are different. An increase in the CD3-CD19+ cell level is an independent risk factor for the deterioration of HRCT imaging. Early intensive treatment with high-dose glucocorticoids can effectively improve imaging prognosis of ILD. Patients with significantly elevated serum ferritin levels should be treated intensively.

## Introduction

Antisynthetase syndrome (ASS) is a rare chronic autoimmune disease characterized by the presence of serum antibodies to aminoacyl-tRNA synthetase (anti-tRNA synthetase antibodies [ASA]) and inflammatory myopathy, interstitial lung disease (ILD), arthritis, fever, Raynaud’s phenomenon, and mechanic’s hands (1). At the 2017 European League Against Rheumatism and the American College of Rheumatology Annual Meeting, ASS should be categorized as a new independent disease separated from other myopathies. To date, at least 10 ASAs have been identified. Anti-histidyl (anti-Jo-1) antibody is the first and most common antibody discovered, followed by anti-threonyl (anti-PL7), anti-glycyl (anti-EJ), anti-alanyl (anti-PL12), and anti-isoleucyl (anti-OJ), while anti-KS, anti-Zo, anti-Yrs, anti-SC, and anti-JS antibodies are less frequently detected (2). Although patients with ASS have common clinical manifestations, previous studies have observed that patients with different ASAs have different clinical features (3, 4).

The lung is the most frequently involved organ and usually manifests as ILD (5). In patients with ASS, ILD is usually dominant at the time of occurrence, and its prevalence is between 63% and 100%, which is considered the main determinant of prognosis (3, 6). There may be differences in the clinical features, laboratory indices, and imaging of patients with ASS-ILD with positive ASA. However, in clinical diagnosis and treatment, the diagnosis time for ASS-ILD is different, the treatment scheme is not standardized, and there are some differences in the prognosis of patients with different subtypes of ASS-ILD. Therefore, there are differences in the prognoses of patients with ASS-ILD. Studies have shown that the prognosis of patients with ASS with positive anti-PL7 and anti-PL12 antibodies is poor, and the survival rate is lower than that of patients with positive anti-Jo1 antibodies (3, 7). Lung involvement is one of the main risk factors for poor survival. Therefore, systematic evaluation of the severity of ILD and follow-up observation of the response to therapeutic drugs are key factors affecting the prognosis of ASS-ILD. Moreover, we hope that through the analysis of survival factors, we can obtain independent risk factors that seriously affect survival time and identify and actively intervene in clinical diagnosis and treatment in advance to improve the survival time of patients. Currently, there are few reports on this topic. Furthermore, there are only a few studies on imaging follow-up and long-term prognosis in ASS-ILD. Therefore, this study systematically reviewed the clinical characteristics of patients with different subtypes of ASS-ILD, evaluated the lung status of patients with ASS-ILD for the first time, provided active drug intervention, followed up the lung imaging changes of patients with ILD, and predicted the factors influencing poor prognosis of ILD on HRCT. At the same time, independent risk factors for survival curves were analyzed.

## Methods

### Study population

This retrospective, single-centered study was conducted at the Yantai Yuhuangding Hospital affiliated with Qingdao University. Consecutive Patients hospitalized between January 2013 and August 2020 were included in this study. ASS-ILD was diagnosed by a multidisciplinary team, including an expert rheumatologist and two experienced radiologists specializing in chest CT. ASS was diagnosed based on the criteria proposed by Solomon et al. (8). Patients with other identifiable causes of ILD were excluded, including medication-related lung injury, malignancy, environmental and occupational exposures. Patients initially diagnosed with heart failure and infectious pneumonia were also excluded.

Demographic data collected from the medical records included age at diagnosis, sex, clinical characteristics at onset, laboratory data at onset, ILD performance at baseline and continuous follow-up, pulmonary function test (PFT), treatment types, ILD results and prognosis, and survival time.

The study was approved by the ethics committee of Yantai Yuhuangding Hospital of Qingdao University (Yantai, China; approval number: 2022-84). Informed consent was waived due to the retrospective study.

### Clinical and laboratory data

All clinical data from the medical charts were obtained during the period from admission to the initiation of treatment. All patients underwent a detailed medical history and physical examination. Blood tests included measurement of hemoglobin (HGB), lactate dehydrogenase (LDH), creatine kinase (CK), C-reactive protein (CRP), ferritin, immunoglobulin (IgG, IgA, IgM, and IgE), lymphocyte subset count, antinuclear antibody (ANA), anti-Ro52 antibody, and anti-tRNA synthetase antibodies (ASAs). Lymphocyte subset counts were detected by Fcm according to the BD Multitest 6-Color TBNK Reagent. ANA was detected by indirect immunofluorescence according to the EU kit. The titer level of ANA ≥ 1:100 indicated that ANA was positive. Using the myositis antibody spectrum kit of OMG (Beijing) Medical Diagnostic Technology Company, the ASAs were identified by EUROIMMUN immunoblot according to the manufacturer’s instructions, including the anti-Jo1, anti-PL7, anti-PL12, anti-EJ, and anti-OJ antibody. And anti-Ro52 antibody was detected by EUROIMMUN immunoblot. The results that showed positive (++) and strong (+++) results were judged as positive, and negative (-) and weak (+) results were judged as negative. PFT data included forced vital capacity (FVC) and carbon monoxide diffusion capacity (DLCO). Values are expressed as percentages of predicted normal values. The restrictive ventilatory dysfunction was defined as a total lung capacity < 80% of the predicted value. The blood gas analysis results were also recorded.

### Imaging data

Lung HRCT images (slice thickness of 1.0 or 1.5 mm) acquired at the first ILD diagnosis and 1 year (at least more than half a year) of follow-up were reviewed. The duration of follow up was noted. Patients who lacked HRCT images before and after treatment in our hospital were excluded. Two radiologists with more than 5 years of experience independently evaluated the lung HRCT images without knowing the clinical information or whether the scans were initial study or follow-up studies and classified the HRCT results according to the 2013 American Thoracic Society classification of idiopathic interstitial pneumonia (9, 10) and the recommendations of the Fleischner Society (11) as follows: usual interstitial pneumonia (UIP), nonspecific interstitial pneumonia (NSIP), organizing pneumonia (OP), NSIP/OP and rapidly progressive ILD (RP-ILD). Follow-up CT images were compared with the initial findings to determine the extent of abnormalities. The ILD course was classified as regression, stability, and deterioration by continuous CT evaluation according to the interpretation of the study radiologists using the method described by Akira et al. (12). Deterioration and regression of the overall ILD range in HRCT images were defined as an increase or decrease of at least 10% of the overall ILD image, whereas stability was defined as changes of less than 10% (12).

### Survival period

All 111 patients diagnosed with ASS-ILD were followed up from enrollment, and the survival time was observed. We all got the ID information and the phone numbers of patients and their guardians at the time of their enrollment, and no follow-up patients were lost.

### Statistical analysis

Normally distributed continuous variables were presented as mean ± standard deviation and compared using analysis of variance (ANOVA). Continuous variables with abnormal distributions were presented as medians (interquartile ranges) and compared between groups using the nonparametric Wilcoxon test. Differences in categorical data were compared using the chi-squared test or Fisher’s exact test. We applied univariate cumulative logistic regression analysis to assess the correlation between each variable of interest and this imaging change. Variables with *p*-values less than 0.1 were subsequently selected for multivariable analysis. Survival plots were generated by applying the Kaplan-Meier product limit method. Cox regression models were used to assess the association between each variable of interest and all-cause mortality during the follow-up period. Variables with p-values < 0.1 were subsequently selected for multivariable analysis. The results are presented as odds ratios (ORs), hazard ratios (HRs), ± 95% confidence intervals (CIs), andp-values. Statistical significance was set at P<0.05. All statistical analyses were performed using Stata (version 14.0; StataCorp LLC, College Station, TX, USA).

## Results

### Clinical characteristics of ASS-ILD

Of the 111 patients with ASS-ILD included in the study, 70 (63.1%) were anti-Jo1-positive, 17 (15.3%) were anti-PL7-positive, 15 (13.5%) were anti-EJ-positive, and 9 (8.1%) were anti-PL12-positive. The general clinical characteristics and comparisons among the 4 groups are shown in **Table 1**. The mean age at onset was 57.0 ± 10.6 years. Most patients were women (Male : Female = 27:84). No differences were observed between the demographic features of the four groups.

**Table 1:** Comparison of demographic and clinical features of patients with ASS-ILD.

Variables	Overall, n = 111	Jo1, n = 70	PL7, n = 17	PL12, n = 9	EJ, n = 15	P value
Age at diagnosis (years)	57 ± 10.64	56.36 ± 10.31	57.59 ± 13.29	57.22 ± 9.91	59.2 ± 9.94	0.552
Female, n (%)	84 (75.68)	54 (77.14)	12 (70.59)	7 (77.78)	11 (73.33)	0.930
Initial clinical characteristics						
Arthritis, n (%)	33 (29.73)	26 (37.14)	4 (23.53)	0 (0)	3 (20.00)	0.083
Dyspnea, n (%)	35 (31.53)	18 (25.71)^*^	5 (29.41)	7 (77.78)^*^	5 (33.33)	0.024
Cough, n (%)	34 (30.63)	11 (15.71)^*^	6 (35.29)	7 (77.78)^*^	10 (66.67)	0.000
Myasthenia/Myalgia, n (%)	21 (19.09)	16 (23.19)	1 (5.88)	1 (11.11)	3 (20.00)	0.439
Mechanic’s hands, n (%)	10 (9.01)	5 (7.14)	3 (17.65)	0 (0)	2 (13.33)	0.400
Raynaud, n (%)	3 (2.70)	1 (1.43)	1 (5.88)	0 (0)	1 (6.67)	0.307
Fever, n (%)	8 (7.21)	6 (8.57)	0 (0)	1 (11.11)	1 (6.67)	0.584

Jo-1, histidyl tRNA synthetase; PL7, threonyl tRNA synthetase; PL12, alanyl tRNA synthetase; EJ, glycyl tRNA synthetase; ^*^P < 0.05 between the groups.

The initial symptoms of patients with ASS-ILD were analyzed. Among 111 patients with ASS-ILD, 28 cases (25.23%) were diagnosed with polymyositis (PM), including 19 cases in anti-Jo1 antibody group, 4 cases in anti-PL7 antibody group, 1 case in anti-pL12 group and 4 cases in anti- EJ group. 47 cases (42.34%) were diagnosed with dermatomyositis (DM), including 29 cases in anti-Jo1 antibody group, 12 cases in anti-PL7 antibody group, 3 case in anti-PL12 group and 3 cases in anti-EJ group. In this study, rash was described in DM patients, including heliotrope rash (8/47, 17.02%), shawl sign (11/47, 23.4.%), V sign (rash on antior neck) (12/47, 25.53%), Gottron papules (40/47, 85.11%), and skin ulcer (2/47, 4.26%). Gottron papules is the most common manifestation of skin lesions in patients with ASS-ILD. Some patients with DM have two or more different forms of rash. The most common first symptom of all patients with ASS-ILD was dyspnea (31.53%), followed by arthritis (29.73%), cough (20.63%), and myasthenia/myalgia (19.09%). The initial clinical manifestations in patients with ASS-ILD with positive ASA in different subtypes were different. Arthritis was the most common first symptom in the anti-Jo1 antibody-positive group, but respiratory symptoms were the most common first symptoms in the other subgroups. Statistical data showed that the incidence of dyspnea was significantly higher in the anti-PL12 antibody group than in the other groups(P=0.024). The incidence of cough as the first symptom in the PL12 and anti-EJ antibody groups was also significantly higher than that in the other groups(P = 0.000).

The comparisons of serological features of patients with ASS-ILD are shown in **Table 2**. The baseline data before treatment showed that the levels of IgG (P=0.006) and IgE (P=0.027) in the anti PL-12 group were higher than those in other subgroups, and the difference was statistically significant. The number of CD16+ CD56+ (NK) cells in the anti-PL7 and anti-PL12 groups decreased significantly, which was statistically significant (P<0.01). There was no significant difference in the positive levels of ANA and anti-Ro52 antibody, CK, CRP, and serum ferritin, as well as in initial oxygen partial pressure (P>0.05).

**Table 2:** Comparison of serological features of patients with ASS-ILD.

Variables	Overall, n = 111	Jo1, n = 70	PL7, n = 17	PL12, n = 9	EJ, n = 15	P value
ANA positive, n (%)	103 (92.79)	65 (92.86)	16 (94.12)	9 (100)	13 (86.67)	0.805
Anti-Ro52^a^ positive, n (%)	53 (74.65)	26 (86.67)	10 (58.82)	8 (88.89)	9 (60.00)	0.066
IgG, g/L	14.5 ± 4.4	13.5 ± 3.6^*^	16.7 ± 4.9	17.5 ± 2.4^*^	15.3 ± 6.2	0.006
IgA, g/L	2.69 (2.01, 3.26)^b^	2.65 (2.00, 3.25)	2.73 (2.19, 3.22)	2.51 (1.96, 4.05)	2.87 (1.95, 3.36)	0.963
IgM, g/L	1.44 (0.95, 2.11)	1.38 (0.92, 2.18)	1.58 (1.23, 2.02)	1.32 (0.94, 1.74)	1.58 (0.9, 2.2)	0.215
IgE, IU/mL	26.1 (15.0, 49.1)	15 (15, 34)^*^	37.1 (25.3, 139.1)	38.8 (27.1, 68.8)^*^	18.1 (15.0, 81.3)	0.027
CK, IU/L	270 (67, 1121)	424.5 (74.3, 1400.3)	388 (63.5, 689)	62 (46, 181.5)	88 (54, 703.3)	0.072
LDH, IU/L	311 (242, 459)	320 (250.3, 542.8)	314 (241, 395)	343 (258.5, 385)	256 (208, 369)	0.266
CRP, mg/dl	6.28 (2.0,19.6)	6.24 (2,15.78)	4.5 (2,19.9)	13.7 (2.7,34.4)	6.22 (2,25.8)	0.759
Serum ferritin, ng/ml	197.3 (108.7, 382)	193.5 (107.9, 278.6)	180.9 (69.9, 399)	470.2 (361.3, 2264.7)	262.4 (97.9, 483.4)	0.060
HGB, g/L	128.3 ± 15.3	127.9 ± 16.6	128.8 ± 10.1	124.8 ± 19.2^*^	131.3 ± 11.2^*^	0.044
PaO_2_, mmHg	77.5 (70.8, 85.8)	78 (71, 89)	77.5 (72.8, 89.4)	67.2 (62.8, 82.8)	81.3 (65.6, 84.6)	0.427
CD3+CD4+, cells/ul	503 (343.5, 730)	501 (364.5, 757)	567 (328.5, 978.8)	323.5 (267.5, 526.8)	597.5 (368, 809.8)	0.187
CD3+CD8+, cells/ul	326 (213.5, 482.5)	385 (236, 504)	269.5 (156.8, 450.5)	237 (110.5, 564)	359.5 (278.3, 604.5)	0.307
CD3-CD19+, cells/ul	180 (107.5, 296)	171 (126.5, 271)	219.5 (46, 460.3)	217 (105.3, 346)	160.5 (51.5, 969.5)	0.832
CD16+CD56+, cells/ul	194 (122, 315.5)	176 (122, 303)	178 (89.8, 328)	134.5 (61.8, 236.5)^*^	279.5 (228, 377.3)^*^	0.003

ANA, antinuclear antibody; IgG,immunoglobulin G; IgA, immunoglobulin A; IgM, immunoglobulin M; IgE, immunoglobulin E; CK, creatine kinase; LDH, lactate dehydrogenase; CRP, C-reactive protein; HGB, Hemoglobin; PaO2, partial pressure of oxygen; CD, cluster of differentiation; Jo-1, histidyl tRNA synthetase; PL7, threonyl tRNA synthetase; PL12, alanyl tRNA synthetase; EJ, glycyl tRNA synthetase; ^*^P < 0.05 between the groups; Anti-Ro52^a^, n=71; ^b^, average value (minimum value, maximum value).

As a whole, the most common type of ILD was NSIP-OP (42 cases, 37.83%), followed by NSIP (38 cases, 34.23%), and OP (25 cases, 22.52%). The incidence of UIP was very low (two cases, 1.80%). The other types of ILD include NSIP+UIP and NSIP+OP+UIP. Moreover, HRCT cannot classify ILD in one patient of anti-PL7 antibody positive. There are some differences in the classification of ILD among different ASS-ILD subtypes (**Table 3**). In the anti-Jo1 antibody-positive group, NSIP-OP was the most common type of ILD (32 cases, 45.71%), followed by OP (16 cases, 22.83%), NSIP (15 cases, 21.43%). Similar results were found in the anti-pL12 antibody positive group, the most common type of ILD was NSIP-OP (5 cases, 55.56%), followed by NSIP (2 cases, 22.22%), and OP (2 cases, 22.22%). However, in the anti-PL7 antibody and anti-EJ antibody positive group, NSIP was the most common type of ILD [anti-PL7, 9 cases, 52.94%; anti-EJ, 9 cases, 60%], followed by OP [anti-PL7, 4 cases, 23.53%; anti-EJ, 3 cases, 20%], and NSIP-OP [anti-PL7, 3 cases, 17.65%; anti-EJ, 3 cases, 20%]. The incidence of NSIP in anti-PL7 and anti-EJ groups was significantly higher than that in anti-Jo1 and anti-PL12 groups (P<0.01). Howere the incidence of NSIP+OP in anti-Jo1 and anti-PL12 groups was higher than that in anti-PL7 and anti-EJ groups(P<0.0). Other ILD types were not statistically different among the groups (P<0.05). And there was no significant difference in FVC% and DLCO% among the groups (P<0.05). Among the patients with ILD, 13 (11.71%) had RP-ILD. The incidence of RP-ILD in the anti-PL12 group was the highest, followed by that in the anti-Jo1 group, although there was no significant difference between the groups (P=0.075).

**Table 3:** Comparison of pulmonary function test and imaging features of patients with ASS-ILD.

Variables	Overall, n = 111	Jo1, n = 70	PL7, n = 17	PL12, n = 9	EJ, n = 15	P value
FVC, %	72.92 ± 15.16	74.34 ± 16.18	76.29 ± 10.06	56.73 ± 13.28	72.19 ± 12.57	0.196
DLCO, %	56.42 ± 16.92	60.00 ± 16.24	51.82 ± 19.75	42.8 ± 15.45	54.33 ± 13.70	0.627
ILD pattern on HRCT						
NSIP, n (%)	35 (31.53)	15 (21.43)^#^	9 (52.94)^#^	2 (22.22)	9 (60)^#^	0.005
OP, n (%)	25 (22.52)	16 (22.83)	4 (23.53)	2 (22.22)	3 (20)	1
NSIP+OP, n (%)	43 (38.74)	32 (45.71)^#^	3 (17.65)^#^	5 (55.56)^#^	3 (20)	0.046
UIP, n (%)	2 (1.80)	2 (2.86)	0 (0)	0 (0)	0 (0)	1
NSIP+UIP, n (%)	4 (3.60)	4 (5.71)	0 (0)	0 (0)	0 (0)	0.845
NSIP+OP+UIP, n (%)	1 (0.90)	1 (1.43)	0 (0)	0 (0)	0 (0)	1
RP-ILD, n (%)	13 (11.71)	9 (12.86)	0 (0)	3 (33.33)	1 (6.67)	0.075
Uncertain type, n (%)	1 (0.90)	0 (0)	1 (5.88)	0 (0)	0 (0)	0.369
HRCT follow-up within 1 year, n (%)	86 (77.48)	53 (75.71)	10 (58.82)	9 (100%)	14 (93.33)	–
Median follow up, (months)	9.64 (6, 12)^*^	9.4 (6, 12)	11 (10, 12)	9.75 (8, 11)	9.4 (8, 12)	–

FVC, forced vital capacity; DLCO, carbon monoxide diffusion capacity; ILD, interstitial lung disease; HRCT, high-resolution computed tomography; NSIP, nonspecific interstitial pneumonia; OP, organizing pneumonia; UIP, usual interstitial pneumonia; RP-ILD, rapidly progressive-interstitial lung disease; GGO, ground-glass opacity; Jo-1, histidyl tRNA synthetase; PL7, threonyl tRNA synthetase; PL12, alanyl tRNA synthetase; EJ, glycyl tRNA synthetase; ^*^, average value (minimum value, maximum value); ^#^P < 0.05 between the groups.

### Analysis on the prognostic factors of imaging progress

A total of 111 patients with ASS-ILD were followed up, and 86 patients (including 53 patients in the anti-Jo1 antibody group, 10 in the anti-PL7 antibody group, 9 in the anti-PL12 antibody group, and 14 in the anti-EJ antibody group) had pre-treatment and post-treatment HRCT imaging data within 1 year (at least more than half a year). The follow-up time of patients in each group was noted. The average duration of the anti-Jo1 antibody group was 9.4 months, the anti-PL7 antibody group was 11 months, the anti-PL12 antibody group was 9.75 months, and the anti-EJ antibody group was 9.4 months (**Table 3**).

**Figure 1:**
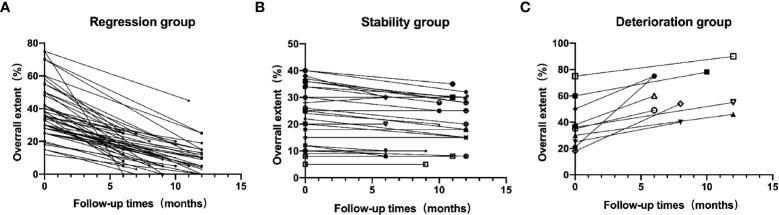
Changes in the overall extent of lung parenchymal abnormalities during follow-up between Regression group **(A)**, Stability group **(B)**and Deterioration group **(C)**.

The patients were divided into three groups according to the imaging changes during ILD follow-up: the regression (49 cases), stability (27 cases), and deterioration (10 cases) groups. Changes in the overall extent of lung parenchymal abnormalities during follow-up between the three groups are shown in **Figure 1**. Specific lesion range values in HRCT findings include the overall extent, and the GGO extent and reticulation extent were in **Table 4**. In the regression groups, 32 cases (32/53, 60.38%) were complicated with anti-Jo1 antibody, 3 cases (3/10, 30%) with anti-PL-7 antibody, 6 cases (6/9, 66.67%) with anti-PL12 antibody, 8 cases (8/14, 87.14) with anti-EJ antibody. In the deterioration groups, 9 cases (9/53, 16.98%) were complicated with anti-Jo1 antibody, 1 case (1/9, 11.11%) with anti-PL12 antibody. Univariate cumulative logistic regression analysis was used to evaluate the correlation between each variable of interest and imaging changes (**Table 4**). Variables with p-values < 0.1 were subsequently selected for multivariable analysis(**Table 4**). The results of this study showed that the initial number of CD3-CD19+ cells in patients with ASS-ILD in the deterioration group increased significantly, while the initial dosage of glucocorticoids in patients in the improvement group was higher. Statistical data showed that the initial number of CD3-CD19+ cells (OR = 1.0013, P=0.014)and the amount of initial glucocorticoid (OR = 0.9603, P = 0.04) were correlated with the outcome of HRCT imaging. The baseline PFT (FVC% and DLCO%), different types of ILD before treatment, and different subtypes of ASA were not significantly different from the outcome of HRCT imaging (P>0.05). A total of 86 patients with ASS-ILD were treated with different immunosuppressants, including oral or intravenous cyclophosphamide (CTX), mycophenolate mofetil (MMF), cyclosporine (CsA), tacrolimus, and azathioprine(AZA). Some patients chose the initial combination treatment of the above two immunosuppressants. However, the choice of immunosuppressants was not related to the outcome of HRCT imaging (P>0.05). Other clinical data and laboratory indices were not found to be correlated with the imaging changes in HRCT (P>0.05).

**Table 4:** Analysis on the prognostic factors of imaging progress in patients with ASS-ILD.

Variables	Total (n = 86)	Regression (n = 49)	Stability (n = 27)	Deterioration (n = 10)	Univariate Analysis			Multivariate Analysis		
					Odds Ratio	95% Conf. Interval	p value	Odds Ratio	95% Conf. Interval	p value
Age, yrs	57 ± 10.64	57.0 ± 10.26	57.3 ± 11.12	56.6 ± 11.61	1.0020	0.9616-1.0403	0.992			
Female, n (%)	69 (80.23)	40 (81.63)	20 (74.07)	9 (90)	0.9516	0.3466-2.6126	0.923			
ANA positive, n (%)	81 (94.19)	48 (97.96)	25 (92.59)	8 (80)	0.1672	0.0296-0.9429	0.043			
RO-52^*^ positive, n (%)	42 (75)	22 (75.86)	14 (73.68)	6 (75)	0.9286	0.2938-2.9351	0.900			
CRP, mg/L	4.6 (2, 15.6)^#^	4.5 (2, 19.1)	4.9 (2, 13.1)	2.6 (2, 23.0)	0.9992	0.9705-1.0287	0.955			
LDH, IU/L	307 (241.8, 391.5)	319 (254, 504)	292 (224, 362)	317.5 (231.8, 457)	0.9990	0.9969-1.0011	0.362			
CK, IU/L	188.5 (62.8, 1055.5)	190 (62.0, 1394.5)	364 (68, 1040)	112.5 (31.5, 1223.0)	1.0000	0.9997-1.0003	0.994			
Serum ferritin, ng/ml	189.3 (92.2, 341.0)	193.7 (109, 372.7)	135.5 (64.8, 305.7)	195.2 (121.2, 1379.0)	1.0002	0.9997-1.0006	0.458			
PaO_2_, mmHg	77 (70.8, 85.6)	76.7 (67.4, 90.6)	77.5 (72.4, 85.0)	78.0 (71.0, 85.3)	0.9925	0.9601-1.0259	0.654			
CD3+CD4+, cells/ul	534 (342, 746)	439 (302.3, 732.3)	585 (472, 714)	650.5 (301.8, 1164.3)	1.0005	0.9991-1.0018	0.502			
CD3+CD8+, cells/ul	367 (244, 536)	352 (204.8, 517.3)	310 (249, 571)	598.5 (424.8, 837.5)	1.0008	0.9985-1.0031	0.505			
CD3-CD19+, cells/ul	177 (107, 292)	147 (84, 224.9)	262 (165, 948)	273 (235.5, 825)	1.0010	1.0000-1.0019	0.046	1.0013	1.0003-1.0023	0.014
CD16+CD56+, cells/ul	194 (121, 311)	190.5 (121.5, 333.5)	222 (72, 249)	156 (127, 493.3)	0.9988	0.9949-1.0027	0.556			
Jo1,n (%)	53 (61.6)	32 (65.3)	12 (44.4)	9 (90)	0.9344	0.4047-2.1572	0.874			
PL7,n (%)	10 (11.6)	3 (6.12)	7 (25.9)	0	1.9367	0.6167-6.0826	0.258			
PL12,n (%)	9 (10.5)	6 (12.2)	2 (7.4)	1 (10)	0.6667	0.1576-2.8192	0.582			
EJ,n, (%)	14 (16.3)	8 (16.3)	6 (22.2)	0	0.8014	0.2674-2.4017	0.693			
RP-ILD,n (%)	10 (11.6)	8 (16.3)	0	2 (20)	0.3786	0.0737-1.9456	0.245			
Baseline FVC, %	73.4 (58.9, 83.8)	68.7 (57.3, 84.8)	74.6 (63.7, 81.9)	74.5 (54.1, 87.9)	1.0040	0.9738-1.0352	0.797			
Baseline DLCO, %	55.2 (44.5, 65.4)	54 (42.4, 66.6)	56.3 (47.8, 63.5)	56.9 (31.3, 72.1)	1.0050	0.9762-1.0347	0.737			
Initial glucocorticoid dose, mg/d	50 (40, 60)	60 (50, 60)	50 (30, 60)	50 (40, 50)	0.9728	0.9486-0.9976	0.032	0.9603	0.9239-0.9981	0.040
HRCT findings										
Initial overall extent, %	33.71 ± 15.84	38.10 ± 15.61	23.85 ± 10.98	38.80 ± 18.03						
Last overall extent, %	20.34 ± 18.7	11.78 ± 9.47	20.37 ± 9.13	62.2 ± 16.34						
Initial GGO extent, %	26.12 ± 17.17	26.86 ± 18.33	20.78 ± 12.21	36.90 ± 19.38						
Last GGO extent, %	19.81 ± 18.67	12.45 ± 10.35	17.93 ± 10.25	61.0 ± 16.31						
Initial reticulation extent, %	6.82 ± 3.35	6.52 ± 3.38	7.26 ± 3.54	6.32 ± 3.13						
Last reticulation extent, %	6.34 ± 2.87	5.84 ± 2.76	7.03 ± 2.94	7.54 ± 4.21						

ANA, antinuclear antibody; CRP, C-reactive protein; LDH, lactate dehydrogenase; CK, creatine kinase; PaO_2,_ partial pressure of oxygen; CD, cluster of differentiation; Jo-1, histidyl tRNA synthetase; PL7, threonyl tRNA synthetase; PL12, alanyl tRNA synthetase; EJ, glycyl tRNA synthetase; RP-ILD, rapidly progressive-interstitial lung disease; FVC, forced vital capacity; DLCO, carbon monoxide diffusion capacity; HRCT, high-resolution computed tomography; GGO, ground-glass opacity; RO-52^*^, n = 56, ^#^, average value (minimum value, maximum value).

### Survival analysis

All 111 patients diagnosed with ASS-ILD for the first time were followed up to observe their survival time. The shortest follow-up time was 6 months, and the longest was 98 months. The five treatment schemes for patients with ASS-ILD were included in this study were as follows: untreated [Survival group, 4/102, 3.92%; Death group, 2/9, 22.22%], anti-fibrosis drugs alone [Survival group, 1/102, 0.98%; Death group, 0], glucocorticoids alone [Survival group, 8/102, 7.84%; Death group, 1/9, 11.11%], hormone-combined immunosuppressants [Survival group, 84/102, 82.35%; Death group, 6/9, 66.67%], hormone-combined immunosuppressants and anti-fibrosis drugs [Survival group, 5/102, 4.9%; Death group, 0]. Nine patients died during follow-up, including seven patients in the anti-Jo1 group (10%), one in the anti-PL7group (5.9%), and one in the anti-PL12 group (11.1%). One patient died of liver failure at the 48th month of follow-up, and the other eight patients died of aggravation of interstitial pneumonia or pulmonary infection.

**Figure 2:**
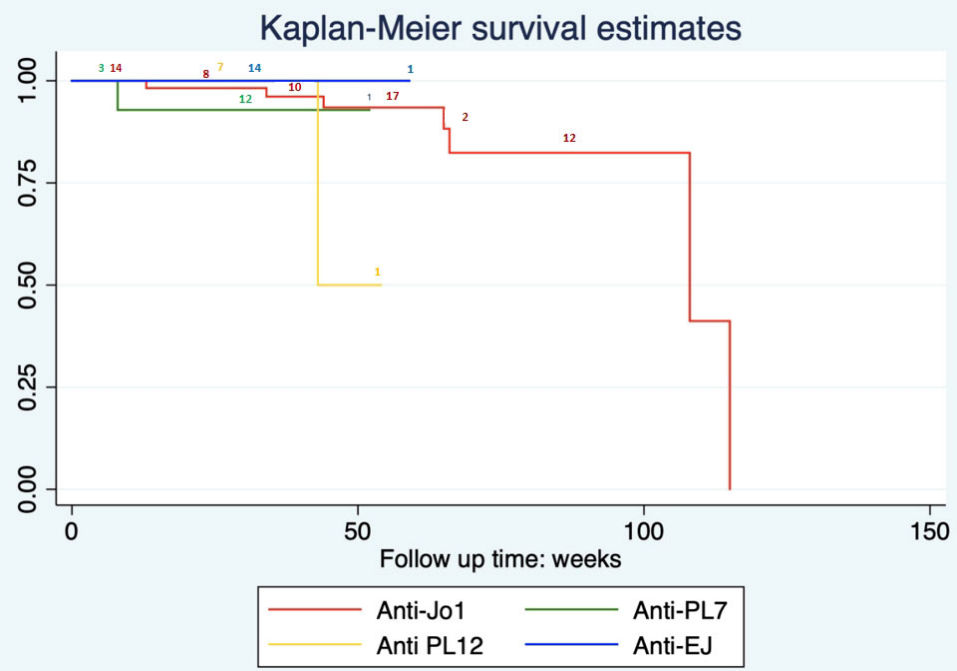
Survival duration of ASS-ILD patients with different ASA antibody positive.

The predictors of adverse outcomes in the survival and death groups were also analyzed (**Table 5**). Survival plots were generated using the Kaplan–Meier product limit method (**Figure 2**). Cox regression models were used to assess the association between each variable of interest and all-cause mortality during the follow-up period. The research data showed that the first symptoms combined with dyspnea (HR=5.8731, P=0.025), severe hypoxemia at diagnosis (HR=0.9196, P=0.025), hypohemoglobinemia (HR=1.0661, P=0.021), elevated serum ferritin level (HR=1.0005, P=0.007), and different treatment regimens (HR=0.5331, P=0.022) were important factors affecting survival time, and there was statistical significance. But, there was no correlation between different ASA subtypes, ILD types, overall extent, GGO extent, baseline FVC% and DLCO% and survival (P>0.05). Variables with p-values < 0.1 were subsequently selected for multivariable analysis. Multivariate analysis showed that only the serum ferritin level was a risk factor affecting survival, and there was a statistically significant difference (HR=1.0007, P=0.049).

**Table 5:** Analysis on the survival factors patients with ASS-ILD.

Variables	Total (n = 111)	Survival (n = 102)	Death (n = 9)	Univariate Analysis			Multivariate Analysis		
				Haz. Ratio	95% Conf. Interval	p value	Haz. Ratio	95% Conf. Interval	p value
Age,yrs	57 ± 10.64	56.3 ± 10.06	64.9 ± 14.18	1.0569	0.9945-1.1232	0.075			
Female, n (%)	84 (75.7)	78 (76.5)	6 (66.7)	0.3007	0.0668-1.3546	0.118			
Initial symptoms									
Arthritis, n (%)	88 (80)	81 (80.2)	7 (77.8)	0.8592	0.5642-1.3085	0.480			
Dyspnea, n (%)	35 (31.5)	31 (30.4)	4 (44.4)	5.8731	1.2467-27.6673	0.025			
Cough, n (%)	34 (30.6)	31 (30.4)	3 (33.3)	3.0260	0.6550-13.9804	0.156			
Myasthenia/Myalgia, n (%)	21 (19.1)	20 (19.8)	1 (11.1)	0.5244	0.0628-4.3774	0.551			
HGB, g/L	128.3 ± 15.3	127.8 ± 15.3	133.3 ± 15.2	1.0661	1.0099-1.1255	0.021	0.9907	0.9214-1.0653	0.802
CK, IU/L	270 (67, 1121)^a^	267 (67.8, 1104.5)	908 (44, 1890)	0.9999	0.9994-1.0004	0.742			
Serum ferritin, ng/ml	197.3 (108.7, 382)	192.7 (107.4, 341.0)	692.5 (264.3, 3281.5)	1.0005	1.0001-1.0009	0.007	1.0007	1.0000-1.0013	0.049
PaO_2_, mmHg	77.5 (70.8, 85.8)	78 (70.9, 86.8)	74.3 (61.0, 83.2)	0.9196	0.8544-0.9897	0.025	1.0034	0.8914-1.1296	0.955
CD3+CD4+, cells/ul	503 (343.5, 730)	518.5 (351, 710.8)	351 (324, 1468)	1.0000	0.9973-1.0028	0.981			
CD3+CD8+, cells/ul	326 (213.5, 482.5)	328 (225, 485.8)	292 (123.5, 569)	1.0002	0.9959-1.0045	0.924			
CD3-CD19+, cells/ul	180 (107.5, 296)	178.5 (89.8, 279.3)	385 (143.5, 713)	1.0008	0.9994-1.0022	0.258			
CD16+CD56+, cells/ul	194 (122, 315.5)	198.5 (121.5, 317.8)	194 (83.5, 409.5)	1.0004	0.9939-1.0071	0.897			
ASA subtypes^b^	-^e^	–	–	1.1947	0.4892-2.9176	0.696			
ILD type^c^	–	–	–	0.7522	0.4495-1.2587	0.278			
RP-ILD, n (%)	13 (11.71)	12 (11.8)	1 (11.1)	9.6894	0.7896-118.9042	0.076			
Overall extent, %	32.26 ± 15.95	33.71 ± 15.93	33.11 ± 23.55	0.958	0.943-1.029	0.491			
GGO extent, %	26.35 ± 15.76	26.06 ± 15.93	29.67 ± 22.20	0.966	0.919-1.015	0.166			
Baseline FVC, %	72.92 ± 15.16	73.05 ± 12.02	55.98 ± 5.78	1.1515	0.947-1.3888	0.140			
Baseline DLCO, %	56.3 (45.8, 65.8)	56.1 (45.9, 67)	57 (37.6, 53.8)	0.9680	0.9018-1.0390	0.368			
Initial glucocorticoid dose, mg/d	50 (40, 60)	50 (40, 60)	60 (50, 60)	0.9987	0.9483-1.0518	0.960			
treatment type^d^	–	–	–	0.5331	0.3109-0.9141	0.022	0.2533	0.0282-2.2722	0.220

HGB, Hemoglobin; CK, creatine kinase; PaO_2_, partial pressure of oxygen; CD, cluster of differentiation; ASA, anti-tRNA synthetase antibody; ILD, interstitial lung disease; RP-ILD, rapidly progressive-interstitial lung disease; GGO,

ground-glass opacity; FVC, forced vital capacity; DLCO,carbon monoxide diffusion Capacity; ^a^, average value (minimum value, maximum value); ASA subtypes^b^, including anti-Jo1 antibody, anti-PL7 antibody, anti-PL12 antibody,

and anti-EJ antibody; ILD type^c^, including NSIP, OP, NSIP+OP, UIP, NSIP+UIP, NSIP+OP+UIP; treatment type^d^, including untreated, anti-fibrosis drugs alone, glucocorticoids alone, hormone-combined immunosuppressants,

hormone-combined Immunosuppressants and anti-fibrosis drugs; -^e^, the statistical software did not output data.

## Discussion

Compared to other inflammatory myopathies, ASS is more likely to be associated with ILD. ILD is the first manifestation in many patients and is easily missed and misdiagnosed (13). Previous data show that patients with ASS have a high incidence of ILD, which is the main cause of morbidity and death (14–16). However, the pathogenesis of ASS-ILD remains unclear. Some studies suggest that the lungs may be the primary affected organ in patients with ASS (17). In susceptible genes, exposure to environmental factors leads to damage to bronchial mucosal epithelial cells (18, 19). Through nonspecific immunization, autoantigens are produced to induce the aggregation of local T and B cells, which causes lung tissue damage by producing specific antibodies (20). However, previous reports on ASS-ILD have a small sample size and lack follow-up data analysis. The overall status of ASS-ILD cannot be fully understood or evaluated. This is the first comprehensive and systematic retrospective study to analyze the clinical characteristics of ASS-ILD, the risk factors for imaging outcomes, and survival factors according to the database.

Previous studies have shown that ASS-ILD is more common in middle-aged and elderly women (5). Our cohort was similar to previous ASS studies in terms of sex distribution and age at diagnosis. The most common symptoms are cough and dyspnea, but all lack specificity. Fever and mechanic’s hands are common, while myositis-related myasthenia is rare. For the clinical manifestations of respiratory system involvement, especially in middle-aged and elderly women with dermatomyositis-related skin changes, such as mechanic’s hands, it is necessary to improve the awareness of ASS-ILD diagnosis. Even if there are no myositis-related manifestations, such as myalgia/myasthenia, screening for anti-synthase antibodies cannot be ignored. As a disease, there are some differences among the ASA subtypes (21). This study not only analyzed the overall clinical, laboratory, and imaging conditions of ASS-ILD patients, but also compared the differences between different ARS-positive ASS-ILD patients to gain a deeper understanding of the overview of ASS-ILD patients. Different subtypes of ASS-ILD also show some heterogeneity in clinical manifestations and hematology. A total of 111 patients with ASS-ILD were included in this study, of which anti-Jo1 was the most common, followed by anti-PL7 and anti-EJ antibodies. Dyspnea and dry cough were the most common initial symptoms, followed by arthritis, which is consistent with previous studies (21). However, there were differences between the subtypes. Arthritis is the most common symptom in patients positive for anti-Jo1 antibody. Therefore, for patients with arthritis complicated by ILD, attention should be paid to screening for ASS. The incidence of respiratory symptoms as the first manifestation of ASS-ILD in patients with positive anti-PL7, PL12 and EJ antibodies was higher. Therefore, for patients with only ILD for the first time, the diagnosis of ASS cannot be ignored when examining the etiology of ILD. This study found that the levels of IgG and IgE in anti-PL12 antibody-positive patients were higher than those in other subgroups before treatment, while the number of CD3+CD4+, CD3+CD8+, and CD16+CD56 + cells decreased significantly compared with other subgroups. However, the number of CD3-CD19+ cells was higher, and the incidence of RP-ILD was higher than that in other subgroups, which was consistent with the previous literature that anti-PL12 positive patients were more likely to develop RP-ILD (3, 7, 21, 22). Some reports emphasize that patients with ASS positive for ant-Jo1 and anti-SSA/Ro antibodies have more severe ILD and a reduced treatment response (23). Our data showed that 74.5% of patients with ASS-ILD were positive for anti-Ro52 antibodies, but no effect on ILD results was found. Whether the coexistence of anti-Ro52 antibody and ass leads to more serious lung diseases requires further study.

Our study found that the baseline FVC% and DLCO% of patients with ASS-ILD with positive ASA in different subtypespatients decreased compared with healthy people. The mean FVC% was less than 80%, which proved to be restrictive ventilatory dysfunction. The decrease of FVC% and DLCO% in the anti-PL12 antibody group was more obvious. Although there was no significant difference, it still provides assistance for clinical diagnosis and treatment. PFT can represent an important prognostic tool for diagnosis and follow up of ILD. Research has found that an FVC% < 60% in Idiopathic Inflammatory Myositis (IIMs) was correlated with a worse prognosis (24). And FVC% seems to be able to predict the response to therapy (25). FVC% and DLCO% also showed correlation at baseline with disease extent on HRCT (26). But Ungprasert et al. Reported a significant correlation was found only for TLC after a 1-year follow up (27). In our study, we did not observe the correlation between FVC% and radiographic outcome and survival factors, and we need to further expand the sample size to explore the value of FVC% in the outcome of ILD and the risk of predicting death. Due to the lack of sufficient specificity or sensitivity of PFT, an HRCT jointly with PFT should be performed when evaluating the severity and follow-up changes of ILD. In this retrospective cohort study, during the follow-up period of 1 year, the majority of ASS-ILD patients lacked the follow-up data on PFT. Therefore, the value of changes in follow-up data for PFT in predicting the outcome of ILD needs to be further confirmed in future studies. In terms of imaging, HRCT of patients with ASS-ILD is characterized by axial, peripheral, and coronal distributions (28). The imaging type of pulmonary interstitial disease is mainly NSIP, followed by NSIP-OP (28). The results of this study showed that NSIP-OP is the most common ILD type of ASS-ILD, followed by NSIP. NSIP-OP is a mixed variant of ILD (29). A typical OP appears in the background of the NSIP. The imaging performance of the NSIP-OP is relatively rare in other ILDs. When imaging NSIP-OP, we should look for the presence of an anti-synthase antibody. At present, there are few studies on imaging follow-up and long-term prognosis of ASS-ILD. In this study, the HRCT imaging findings of patients with ASS-ILD were followed up for 1 year, and survival was followed up for a long time.

In addition, this study revealed potential risk factors for HRCT image deterioration in patients with ASS-ILD. It was found that the initial number of CD3-CD19+ cells and initial glucocorticoid dosage were correlated with imaging progression. At the same time, this study found that an increase in the number of B lymphocytes before treatment, whether using univariate or multivariate analysis, was an independent risk factor for the deterioration of ASS-ILD, which also provides a basis for biologically targeted drugs to eliminate B lymphocytes for the treatment of refractory ASS-ILD. However, this study did not re-compare the lymphocyte counts at the end of imaging follow-up in patients with ASS-ILD. B-lymphocytes play an important role in the pathogenesis of rheumatic diseases. An increasing number of studies have shown that clearing B cells can be used to treat a variety of refractory autoimmune diseases (30, 31). Krystufkova et al. (32) observed 99 patients with ASS and found that the level of B-cell stimulating factor (BAFF) in the serum of patients with ILD was higher than that of patients without ILD (P<0.05). Another study found that the serum BAFF level of dermatomyositis (DM)/polymyositis(PM)patients with positive anti-Jo1 antibody was positively correlated with the titer of anti-Jo1 antibody, and the fluctuation of BAFF levels could indicate the activity of myositis (33). The results showed that the B-cell analysis pathway may be involved in the pathogenesis of this type of disease. On the other hand, it also suggests that targeted killing of B cells can be used as a treatment method for patients with ASS-ILD. Marie et al. (34) reported that seven patients with refractory ASS-ILD were followed up for 12 months after receiving rituximab (RTX) treatment and found that FVC/DLCO increased and a decreased/stabilized in the extent of ILD. A retrospective study showed that the PFTs of 24 RTX-trarted ASS with severe ILD patients were improved after a median 52 months follow up (35). Due to retrospective clinical studies, the patients with ASS-ILD enrolled in this study were not treated with RTX, combined with the results of this study and the data of previous studies, it provides a basis for our follow-up treatment of refractory ASS-ILD. In the future, the relationship between CD3-CD19+ and deterioration needs to be further confirmed by increasing the sample size.

Some studies have reported that the mode of ILD (such as UIP) is an independent risk factor for the poor prognosis of ILD (36); however, this study did not draw this conclusion, and is considered to be related to the very low incidence of UIP in the mode of ASS-ILD. Previous reports on ASA and prognosis have indicate that various types of ASA can predict different prognosis (7, 37). Positive anti-PL7 and anti-PL12 antibodies are associated with a poor prognosis of ILD (38). In the long-term follow-up, the prognosis of anti-EJ antibody patients was better than that of other patients, but the survival rate of anti-PL7 antibody patients decreased faster in the early stage than in the late stage (3). However, we found that there is no correlation between the ASA subtype and ILD prognosis in the univariate and multivariate analyses. Love et al. (22) found that the poor prognosis of ASS seems to be unrelated to ASA subtypes, but to the high incidence, severity, and steroid resistance of ILD. We believe that this view is reasonable. As this was a retrospective study, larger prospective studies are needed to evaluate the possible association between ASA subtypes and Prognosis of ASS-ILD in the future.

This study also analyzed the survival factors of ASS-ILD patients. In the univariate analysis, dyspnea, decrease in HGB, increase in serum ferritin titer, decrease in oxygen partial pressure, and treatment type were the risk factors for predicting death in patients with ASS-ILD. The level of serum ferritin in the ASS-ILD death group was significantly higher than that of survival patients, which was an independent risk factor for predicting survival, whether univariate or multivariate analysis. As an acute-phase protein, serum ferritin plays an important role in the storage and circulation of iron metabolism, as well as the immune response of the host. Moreover, its level was significantly higher in patients with inflammatory diseases, autoimmune diseases, chronic infections, and malignancies. Shi J et al. (2) found that according to survival analysis, RP-ILD and high serum ferritin are indicators of poor prognosis in patients with anti-synthase syndrome. This was similar to our results. However, our study did not find that RP-ILD was associated with the risk of death, which needs to be further confirmed by increasing the sample size in the follow-up. At the same time, their results also showed that serum ferritin was significantly increased in patients with ASS complicated with RP-ILD. Gono et al. (38) reported that patients with DM/CADM with increased ferritin levels showed a higher incidence of RP-ILD, and the survival rates of patients with higher ferritin levels were significantly lower than those with low ferritin levels. The results showed that the level of serum ferritin not only conferred a poor outcome, but also can be related to the disease activity of ILD. Therefore, for patients with ASS-ILD with significantly increased serum ferritin levels, the initial treatment should be strengthened and close follow-up observation should be conducted to improve the survival rate of patients. Study has found that the initial HRCT diagnosis may affect the long-term survival rate of patients, especially when chronic lesions such as Honey combing and Fibrosis exist in the early stage of the disease (12). No correlation between the initial HRCT findings and mortality rate was found in this study. For ASS-ILD patients, there are many factors affecting the prognosis of patients in the long-term follow-up treatment process, including patients’ self-withdrawal of drugs, long-term immunosuppressive therapy leading to infection, which will affect the survival rate of patients. Therefore, follow-up prospective studies should be carried out to explore the correlation between HRCT initial findings and survival in ASS-ILD patients.

This study had several limitations. First, the number of non-anti-Jo1 antibody patients and deaths included in the study was relatively small, and the sample size needs to be expanded in the future. Second, due to the small number of patients in the non-Jo1 antibody group, this limits the analysis of confounding factors. Third, the diagnosis of ILD lacks histological evidence. And due to the retrospective nature of the study, follow-up data for FVC% were lacking.

## Conclusion

Our study showed that in patients with ASS-ILD with dyspnea and cough as the first symptoms, the levels of IgG and IgE and the number of CD16+ CD56+ (NK) cells were significantly different among the four different ASA groups. The number of CD3-CD19+ cells and the initial glucocorticoid dosage were correlated with imaging progress, and were independent risk factors for ILD deterioration. Dyspnea as the first symptom, hypohemoglobinemia, serum ferritin level, oxygen partial pressure at diagnosis, and different treatment types were important factors affecting survival, and serum ferritin level was an independent risk factor for survival. Knowing the predictors of ILD imaging progress in patients with ASS is important for the early rational management of disease and improving prognosis. However, further prospective series studies are needed to better determine the risk factors for long-term deterioration of ILD and conduct clinical validation in the future.

## Data availability statement

The original contributions presented in the study are included in the article/supplementary material. Further inquiries can be directed to the corresponding author.

## Ethics statement

The studies involving human participants were reviewed and approved by the ethics committee of Yantai Yuhuangding Hospital of Qingdao University (Yantai, China; approval number: 2022-84). Written informed consent for participation was not required for this study in accordance with the national legislation and the institutional requirements.

## Author contributions

NZ, YL, and WJ conceived the idea for the study. HW conducted the statistical analysis. PW and XW managed the imaging data. YB, YL, and YT collected clinical data. NZ drafted the manuscript with inputs from all co-authors. All authors have reviewed and approved the final manuscript.

## Conflict of interest

The authors declare that the research was conducted in the absence of any commercial or financial relationships that could be construed as a potential conflict of interest.

## Publisher’s note

All claims expressed in this article are solely those of the authors and do not necessarily represent those of their affiliated organizations, or those of the publisher, the editors and the reviewers. Any product that may be evaluated in this article, or claim that may be made by its manufacturer, is not guaranteed or endorsed by the publisher.
